# The Better Times Now

**Published:** 1901-07

**Authors:** 


### THE BETTER TIMES NOW.

THE return of better prices in live-stock, especially in equine values, has wrought many changes along veterinary lines that are very gratifying to the profession. It has given renewed interest to all matters connected with association progress and legislative enactments of direct and indirect importance. It has increased the interest of the individual members in their own calling because of increased recognition and remuneration for their services. It has added materially to the welfare of the colleges by increased classes, and the trying times have brought a better class of students to their doors. The unsupplied demand of the Bureau of Animal Industry for additional inspectors is a welcome sign of the times, and the greater disposition and tendency to enter private practice or remain at the latter post because of the better returns has not been overcome by the increased salaries granted by our Government. The most trying period of our profession has passed, and in welcoming the better times and situations we should not be unmindful of the added responsibilities in the guidance of our schools, the better regulations for practice, the restraint of unwise and dangerous breeding, and the passage of proper laws and wise sanitary police measures.

